# A Systematic Multi-Dataset, Multi-Seed Evaluation of Preprocessing Strategies for Retinal Optic Disc and Cup Segmentation

**DOI:** 10.3390/diagnostics16172880

**Published:** 2026-09-07

**Authors:** Abdullah Alajmi, Youssef Elnahal, Mohamed Othman, Manal Aljuhani, Amani Alharbi, Ghada Abdelhady

**Affiliations:** 1 Department of Computer Science, College of Computer Engineering and Sciences, Prince Sattam Bin Abdulaziz University, Al-Kharj 11942, Saudi Arabia; a.alajmi@psau.edu.sa; 2 Electrical Systems Engineering Department, October University for Modern Sciences and Arts (MSA University), 6th of October City 12572, Egypt; youssef.waleed4@msa.edu.eg; 3 Computer Systems Engineering Department, October University for Modern Sciences and Arts (MSA University), 6th of October City 12572, Egypt; malsafy@msa.edu.eg (M.O.); gabdelmouez@msa.edu.eg (G.A.); 4 Department of Radiology and Medical Imaging, College of Applied Medical Sciences, Prince Sattam Bin Abdulaziz University, Al-Kharj 16278, Saudi Arabia; ama.alharbi@psau.edu.sa; 5 Radiological Sciences and Technology Research Unit, College of Applied Medical Sciences, Prince Sattam Bin Abdulaziz University, Al-Kharj 16278, Saudi Arabia

**Keywords:** CLAHE, deep learning, EfficientUNet++, fundus images, glaucoma detection, image preprocessing, multi-seed evaluation, optic cup segmentation, optic disc segmentation, reproducibility, ablation study

## Abstract

**Background/Objectives:** Accurate delineation of the optic disc and optic cup in retinal fundus photographs is a prerequisite for automated glaucoma screening. While encoder–decoder segmentation models have advanced considerably, the contribution of upstream preprocessing to segmentation accuracy, and the stability of that contribution across repeated training runs, remain insufficiently characterized. **Methods:** Five preprocessing pipelines, baseline, Contrast Limited Adaptive Histogram Equalization (CLAHE), Region of Interest (ROI) cropping, ROI+CLAHE, and CLAHE with heavy augmentation, were benchmarked under a fixed EfficientUNet++ model with an EfficientNet-B7 encoder on three publicly available fundus datasets (REFUGE, ORIGA, and Drishti-GS). Every configuration was retrained under three independent random seeds (42, 15, and 89) to assess run-to-run variability. Seed-level standard deviations accompany every reported mean and define the confidence limit on each ranking. **Results:** On REFUGE, CLAHE with augmentation (Config 5) achieved the strongest mean Dice (disc 0.9523±0.0017; cup 0.8348±0.0018). On ORIGA, all five configurations clustered within 0.0067 disc Dice; ROI+CLAHE (Config 4) was marginally ahead on disc (0.9681±0.0002) and augmentation led on the cup (0.8873±0.0024). On Drishti-GS, all five configurations converged successfully once optimizer and loss settings were corrected; the near-total failures seen in earlier single-run experiments reflected a configuration problem, not the small (81-image) training set. **Conclusions:** CLAHE applied to full-resolution images is the single most consistently beneficial preprocessing choice across all three datasets. ROI+CLAHE showed a small, initialization-stable advantage on ORIGA, but ROI crop centres were derived from ground-truth centroids, an oracle localization setting, and these results should not be interpreted as achievable by a fully automated pipeline. Data augmentation showed a consistent reduction in initialization sensitivity on small datasets and may be beneficial as a default strategy.

## 1. Introduction

According to recent estimates, there are around 80 million people who have been diagnosed with glaucoma at present, with the increasing rate expected to reach 111.8 million by 2040 [1]. Glaucoma is one of the leading causes of irreversible blindness in the world. The cup-to-disc ratio (CDR) is one of the important diagnostic indicators for glaucoma. CDR measures the area of the optic cup in relation to the area of the disc [2]. To obtain CDR reliably from fundus photographs, it is necessary to segment the optic cup and disc accurately. The process of automating segmenting when measuring CDR can help increase screening in remote areas where there is little or no access to medical specialists.

Nonetheless, achieving reliable segmentation is not straightforward. Specialists must face (1) the inter-subject variations in disc morphology; (2) the lighting issues and different camera qualities; (3) the soft nature of cup boundaries with varying positions even among ophthalmologists; and (4) the interference of the retinas’ blood vessels in any texture information [3].

Encoder–decoder networks, pioneered by U-Net [4] and its successors, have spearheaded significant advancement in tackling these challenges. However, upstream preprocessing, as well as the question of whether a single training run can produce indicative rankings during repeated experiments, remain largely underexplored within the literature. To our knowledge, no research has previously conducted a systematic comparison of preprocessing setups for optic disc and cup segmentation using a multi-seed reproducibility protocol in combination with the controlled isolation of preprocessing across multiple public benchmarks, which produces proof of ranking stability that single-run ablation studies cannot provide.

### 1.1. Research Gap

The utilization of techniques such as CLAHE [5] and region-of-interest (ROI) cropping are commonly integrated into fundus analysis pipelines. However, few studies have isolated and evaluated their individual and combined contributions under controlled experimental conditions. In addition, most existing studies fail to evaluate the consistency of method rankings across repeated executions of the same experiment, even though these rankings may fluctuate due to experimental variability. Papers typically vary preprocessing and architecture together, so a gain of one or two Dice points cannot be assigned to either. Single-run evaluation on a single dataset compounds the problem: with one seed and one benchmark there is no way to separate a real effect from a lucky initialization.

Recent work has pushed in two directions: federated training across clinical sites and hybrid models that place a transformer encoder behind CLAHE-based contrast normalization [6,7]. Both report gains. Neither separates the contribution of the contrast step from that of the architecture, because both were changed at once. Nor has either been repeated across seeds on more than one benchmark, which is what it takes to tell a preprocessing effect from initialization noise.

### 1.2. Contributions

Against that background, our contributions are:

1. A five-way ablation under a frozen architecture. Encoder, decoder, optimizer, schedule, and epoch budget are identical in every run; only the preprocessing changes. ROI extraction, CLAHE, and augmentation can therefore be read separately.
2. Three seeds per cell. Every configuration, dataset pair was trained three times, under seeds 42, 15, and 89. We report mean and sample standard deviation, so a reader can see which rankings survive reinitialization and which do not.
3. Three benchmarks, one protocol. REFUGE, ORIGA, and Drishti-GS span 400, 650, and 101 images and 10.0%, 25.8%, and 69.3% glaucoma prevalence. Running the same protocol on all three shows where a preprocessing recommendation holds and where it does not.
4. An explicit statement of what the ROI numbers mean. Crop centres come from ground-truth mask centroids, not from a detector. This keeps localization error out of the comparison and it also caps how much the ROI results can claim; we treat them as dataset-conditional rather than general.

## 2. Related Work

### 2.1. Deep Learning for Optic Disc and Cup Segmentation

U-Net [4] set the pattern for dense medical segmentation: skip connections carry encoder features across to the decoder so that spatial detail survives downsampling. UNet++ [8] densified those connections into nested pathways, narrowing the semantic distance between the features being joined. Backbone choice evolved in parallel. EfficientNet [9] showed that scaling depth, width, and input resolution together beats scaling any one of them alone, which makes its ImageNet weights an economical starting point when labelled medical data are scarce.

Later work tried to make the network attend selectively. Attention U-Net [10] gates each skip connection so that irrelevant activations are damped before they reach the decoder. Wang et al. [11] took a different route and cast segmentation as adversarial learning in output space, which sharpened predicted boundaries. Fu et al. [12] built M-Net around a multi-scale input and a polar transformation that re-expresses the problem in coordinates matched to the roughly annular geometry of the disc and cup; they reported gains on ORIGA and REFUGE.

Transformers have since entered the field. Hussain and Basak [13] inserted cross-attention between the encoder levels of U-Net (UT-Net) and reported strong disc and cup Dice on REFUGE and Drishti-GS. Kumar and Kumar [14] combined dual-attention decoder modules with dilated dense skip convolutions and added a vessel-inpainting stage upstream of segmentation, reaching 0.9595 disc and 0.8870 cup Dice under five-fold cross-validation.

### 2.2. Preprocessing in Medical Imaging

CLAHE [5] equalizes within small tiles rather than over the whole image and clips the transfer function, so local contrast rises without the noise amplification that global histogram equalization produces. Tan et al. [15] measured clear gains for vessel segmentation but also found over-enhancement on images that were already well exposed. That asymmetry is the reason contrast preprocessing has to be tested per dataset rather than assumed.

A 2025 survey [16] finds CLAHE still the dominant contrast method in fundus pipelines. Ubiquity of that kind is precisely what makes an isolated, a multi-seed measurement of its contribution worth having.

ROI cropping is the other habitual step. Sevastopolsky [17] showed that cropping around the disc removes background clutter and lets the network spend its capacity on the structure of interest. What such reports frequently leave unsaid is whether the crop centre came from a ground-truth mask or a detector. The omission matters, because only the second case tells you anything about unseen images.

Augmentation compensates for small corpora by adding geometric and photometric variation. How much it helps depends on the dataset and on the rest of the training setup. Small-corpus models are also the ones most sensitive to initialization, which is an argument for measuring augmentation across several runs rather than one.

### 2.3. Gap Analysis

Taken together, the literature reveals a recurring blind spot: preprocessing pipelines are almost always treated as fixed, unjustified design choices rather than variables to be ablated. Disentangling the contribution of data preparation from that of the network architecture requires holding one constant while varying the other, a design that is rarely implemented. Equally missing is evidence that the observed performance rankings are stable across repeated training runs; without such evidence, apparent advantages of one preprocessing strategy over another may simply reflect random variation in weight initialization. Coverage of multiple datasets under a single controlled protocol is also unusual, and authors seldom clarify whether ROI coordinates originate from ground-truth annotations or an automated localization step, an omission that makes it impossible to assess the practical deployability of reported ROI-based results.

## 3. Materials and Methods

### 3.1. Datasets

We evaluated preprocessing configurations on three benchmark datasets with deliberately diverse characteristics, summarized in Table 1.

REFUGE contains 400 training images with high image quality and consistent illumination. ORIGA comprises 650 fundus images with greater quality variability and higher glaucoma prevalence. Drishti-GS contains 101 images with diverse acquisition characteristics and annotation uncertainty from four independent ophthalmologists, making it the most challenging dataset in this study by virtue of its small size and annotation heterogeneity.

All experimental results for the REFUGE, ORIGA, and Drishti-GS datasets, covering all five preprocessing configurations and three independent random seeds (42, 15, and 89), are publicly available in the Zenodo repository (DOI: https://doi.org/10.5281/zenodo.21416763).

### 3.2. Preprocessing Configurations

We evaluated five configurations maintaining identical network architecture, training procedures, and hyperparameters to isolate preprocessing effects. Table 2 summarizes the experimental matrix.

Configuration 1 (Baseline): Full-resolution images resized to 512×512 pixels, normalized to [0,1] and standardized using ImageNet statistics (mean = [0.485, 0.456, 0.406], std = [0.229, 0.224, 0.225]).

Configuration 2 (Full-Size + CLAHE): CLAHE applied prior to resizing (clip limit = 8.0, tile grid = 8×8), enhancing local contrast across the full retinal field.

Configuration 3 (ROI Extraction): A square region of 512×512 pixels is extracted around the centroid of the bounding box of the ground-truth disc/cup mask for each image and used in place of the full-resolution image. The ROI centre is therefore determined from the available ground-truth annotation rather than predicted by an automated disc-localization model. This design was adopted to isolate the effect of ROI-based spatial focusing on segmentation performance without introducing localization errors as an additional experimental variable. Consequently, the reported ROI results evaluate segmentation under idealized localization conditions and should not be interpreted as the performance of a fully automated end-to-end pipeline; see Section 6 for further discussion.

Configuration 4 (ROI + CLAHE): Combines the ROI extraction described above with CLAHE enhancement (clip limit = 8.0, tile grid = 8×8) applied within the cropped region.

Configuration 5 (Full-Size + CLAHE + Augmentation): Extends Config 2 with data augmentation: horizontal and vertical flipping (*p* = 0.5), a randomly selected grid or optical distortion (*p* = 0.7), shift–scale–rotate (scale ±0.2, rotate $\pm \;30^{\circ }$, *p* = 0.5), random brightness/contrast and gamma adjustment (*p* = 0.5 each), followed by CLAHE and normalization. The effective dataset size is multiplied 4× through this augmentation pipeline.

All experimental results for the REFUGE, ORIGA, and Drishti-GS datasets, covering all five preprocessing configurations and three independent random seeds (42, 15, and 89), are publicly available in the Zenodo repository at https://doi.org/10.5281/zenodo.21416763.

### 3.3. Network Architecture

**Figure 1:**
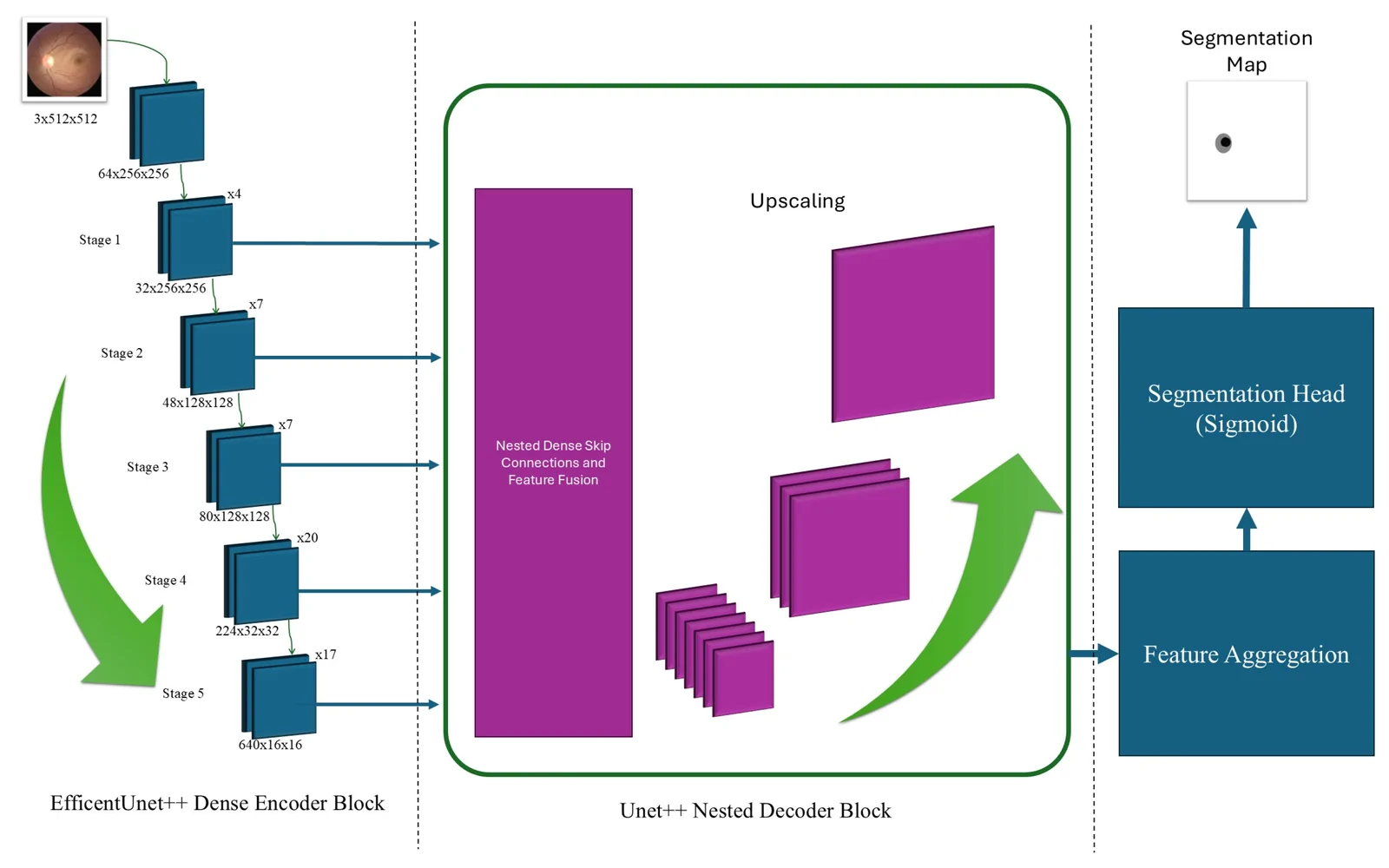
EfficientUNet++ architecture used in this study. The encoder is EfficientNet-B7 pretrained on ImageNet with compound scaling factor φ = 2.0. The decoder follows the UNet++ design with dense nested skip connections at each resolution level. The segmentation head produces a three-channel softmax output representing background, optic cup, and optic disc classes, respectively.

We employed EfficientUNet++, combining a UNet++ decoder with an EfficientNet-B7 encoder pretrained on ImageNet. The architecture is illustrated in Figure 1.

The encoder applies compound scaling across depth, width, and resolution simultaneously, yielding approximately 66 M parameters. Total network parameters: approximately 66.4 M (66.0 M encoder, 0.4 M decoder).

Table 3 summarizes the EfficientNet-B7 encoder stage specifications and their corresponding UNet++ decoder connections.

Justification for EfficientNet-B7: EfficientNet-B7 was selected over lighter backbones (e.g., B0–B3) because compound scaling of depth, width, and input resolution together yields favourable accuracy-per-parameter trade-offs relative to scaling any single dimension in isolation [9], and because ImageNet-pretrained EfficientNet backbones transfer effectively to medical imaging tasks with limited labelled data, as is the case for Drishti-GS (81 training images). A systematic backbone-size ablation was not performed and is noted as future work in Section 6.

### 3.4. Training Procedure

Loss Function: A combined Dice loss and Cross-Entropy loss with equal weights:

(1) $$L=0.5\;L_{\mathrm{CE}}+0.5\;L_{\mathrm{Dice}}$$

where the Dice loss for class *c* is:

(2) $$L_{\mathrm{Dice}}^{\left(c\right)}=1-\frac{2\sum_{i}p_{i}^{\left(c\right)}\;g_{i}^{\left(c\right)}+\varepsilon }{\sum_{i}{\left(p_{i}^{\left(c\right)}\right)}^{2}+\sum_{i}{\left(g_{i}^{\left(c\right)}\right)}^{2}+\varepsilon }$$

computed jointly over the cup and disc classes (background excluded from the Dice term).

Optimization: AdamW optimizer with learning rate η = $10^{-4}$, weight decay λ = 0.01. Cosine annealing learning rate schedule (CosineAnnealingLR, $T_{\max}=100$). Training for 100 epochs with batch size 5 and gradient accumulation over 8 steps (effective batch size 40). Mixed precision training (AMP) via torch.cuda.amp with automatic gradient scaling.

Model Selection: The checkpoint achieving the highest mean validation Dice coefficient averaged across the optic disc and optic cup classes, evaluated on the fixed validation split at the end of each epoch, is retained. No test-time augmentation (TTA) was applied; all reported Dice and Intersection over Union (IoU) values reflect single-pass inference on unaugmented validation images. Training and validation loss curves for all configurations and seeds are provided in the Zenodo data archive at https://doi.org/10.5281/zenodo.21416763 and provide smooth convergence within the 100-epoch budget for every configuration.

### 3.5. Reproducibility Protocol: Multi-Seed Training

Every configuration was trained independently on each dataset using three random seeds (42, 15, and 89), affecting weight initialization, data shuffling, and augmentation sampling. For each configuration, we report the mean and sample standard deviation of the Dice coefficient across the three runs to evaluate the stability of the observed performance rankings. Accordingly, when the difference in mean performance between configurations is small relative to the observed variability across seeds, the resulting ranking should be interpreted with caution.

Formal per-image statistical significance testing was not performed. The evaluation loop computed both running batch-level aggregates and individual per-image Dice scores during evaluation, with the per-image scores retained in the TensorBoard logs. Statistical testing across the three random seeds was likewise not performed because the limited number of runs provides insufficient statistical power; with n=3, an exact two-sided Wilcoxon signed-rank test cannot yield p<0.05 under any arrangement of the data. The mean ± standard deviation (SD) values below therefore describe run-to-run spread and indicate the direction of ranking differences. Three seeds provide a practical indicator of run-to-run variability but constitute a small sample; therefore, the observed performance trends should be interpreted as indicative rather than definitive rankings.

### 3.6. Evaluation Metrics

Dice Similarity Coefficient (DSC):

(3) $$\mathrm{Dice}=\frac{2|P\cap G|}{|P|+|G|}$$

Intersection over Union (IoU/Jaccard Index):

(4) $$\mathrm{IoU}=\frac{|P\cap G|}{|P\cup G|}$$

Dice is calculated per class (cup, disc) at the best validation epoch for each run and then averaged across the three seeds.

Equation (4) gives the standard definition of IoU computed directly from per-image predictions and intersections. In the present study, because the evaluation pipeline retains only batch-averaged Dice (see Section 3.5), the IoU values reported in Table 4, Table 5 and Table 6 are approximations derived from the mean Dice score using the algebraic identity IoU=Dice/(2−Dice). These derived values are marked with a dagger (†) in all tables to distinguish them from independently computed IoU.

## 4. Results

### 4.1. Overall Performance Comparison

Table 4, Table 5 and Table 6 report mean ± SD across the three training seeds for each configuration and dataset.

### 4.2. REFUGE Dataset Results

**Figure 2:**
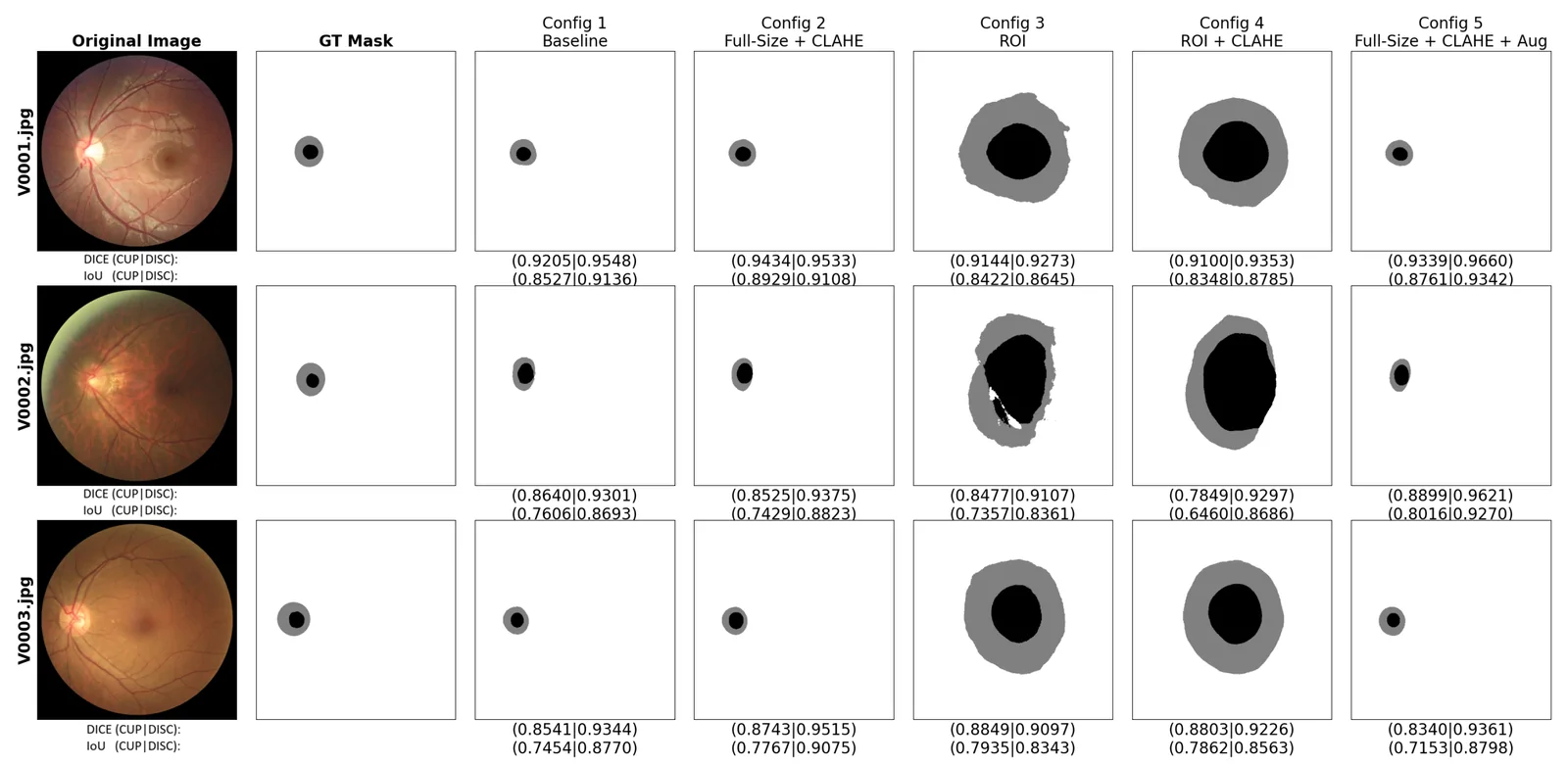
REFUGE validation image V0001.jpg under all five configurations, seed 42. Dice values printed here belong to that single seed; three-seed means and standard deviations are in Table 4. Config 2 and Config 5 give the cleanest disc and cup boundaries of the five.

On the REFUGE benchmark, Configs 5 and 2 emerged as the top-performing preprocessing strategies. Config 5 (Full+CLAHE+Aug) attained a mean disc Dice of 0.9523±0.0017 and mean cup Dice of 0.8348±0.0018, narrowly ahead of Config 2 (Full+CLAHE) at 0.9465±0.0038 and 0.8230±0.0050, respectively. Importantly, both configurations exhibited tight seed-to-seed variation (disc and cup SD ≤0.005), indicating that the benefit of full-image CLAHE is not an artefact of a favourable weight initialization but a consistent effect across all three seeds. Spatial cropping (Configs 3 and 4) produced lower scores overall; Config 3 in particular showed the widest seed spread (disc SD = 0.0107), which aligns with the reduced contextual information available to the network when only the cropped disc region is presented without accompanying contrast normalization. Qualitative predictions for all five configurations are shown in Figure 2.

### 4.3. ORIGA Dataset Results

**Figure 3:**
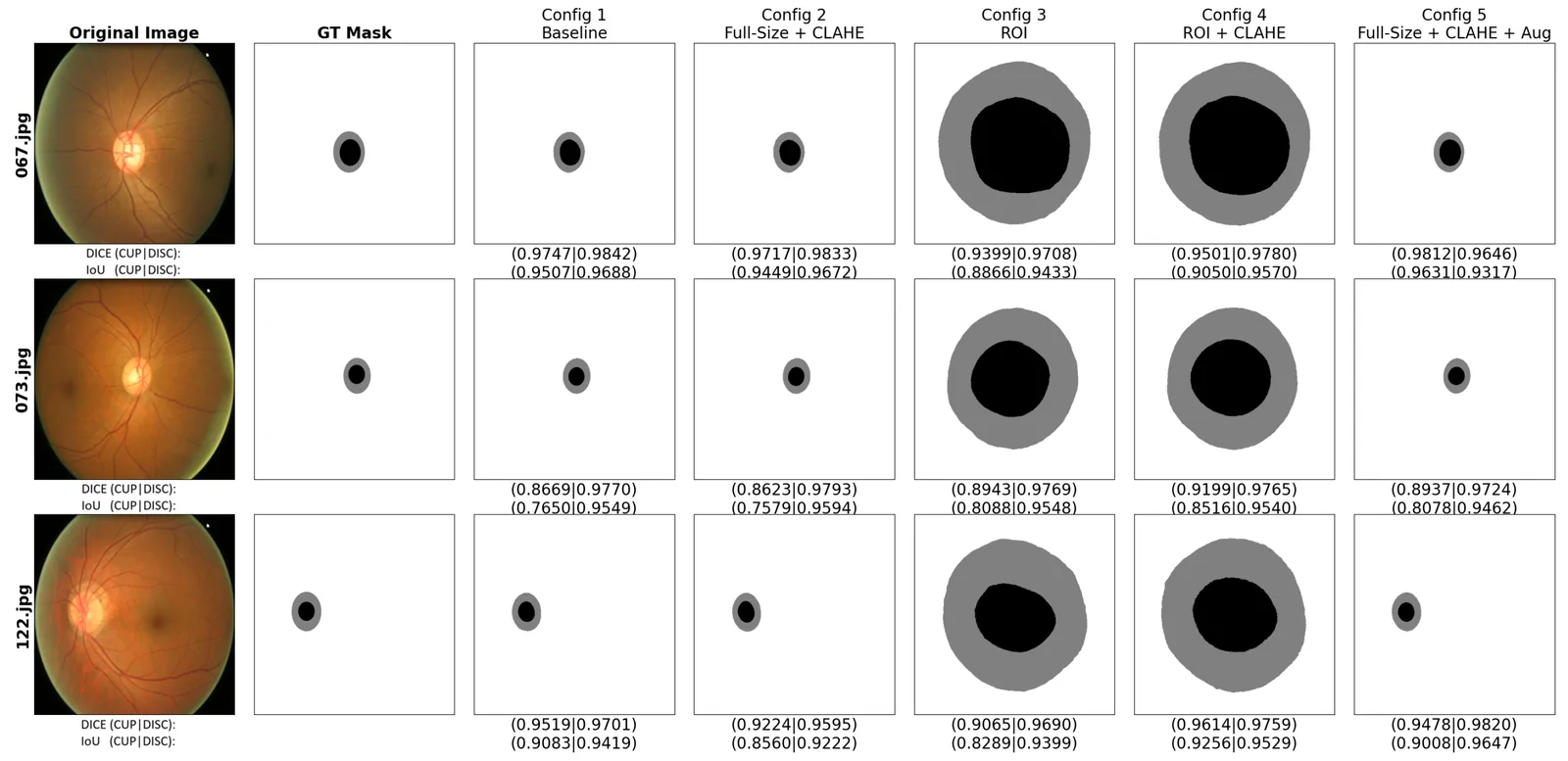
Qualitative segmentation results on representative ORIGA validation images (seed 42) for Config 3 (ROI, Ep. 98) and Config 5 (Full+CLAHE+Aug, Ep. 31), the two configurations for which prediction outputs at the best validation checkpoint were retained for this seed. Quantitative performance for all five configurations, including Configs 1, 2, and 4 (not shown qualitatively here due to checkpoint retention limits), is fully reported in Table 5. Annotated Dice values reflect this representative seed.

ORIGA produced a different picture. All five configurations landed high and close together: disc Dice ran from 0.9614 to 0.9681, a span of 0.0067, and seed-level standard deviations for the disc stayed at or below 0.0017 in every cell. A 520-image training partition buys that stability. Inside the cluster, Config 4 (ROI+CLAHE) took the disc at 0.9681±0.0002 with Config 3 just behind at 0.9666±0.0003, and Config 5 took the cup at 0.8873±0.0024. These margins are a few ten-thousandths of a Dice point. Visual predictions for representative ORIGA samples are provided in Figure 3.

### 4.4. Drishti-GS Dataset Results

Under the multi-seed evaluation protocol and corrected training configuration used in this study, all five preprocessing strategies, including the unaugmented baseline, converge to meaningful segmentation accuracy (combined Dice+CE loss, ImageNet-pretrained encoder, AdamW with $\eta =10^{-4}$). This confirms that, once the loss function and initialization scheme are appropriately chosen, unaugmented training on Drishti-GS’s 81 images does not inherently fail; the near-total failures observed in preliminary single-seed experiments with an uncorrected setup were attributable to training configuration sensitivity rather than data scarcity.

**Figure 4:**
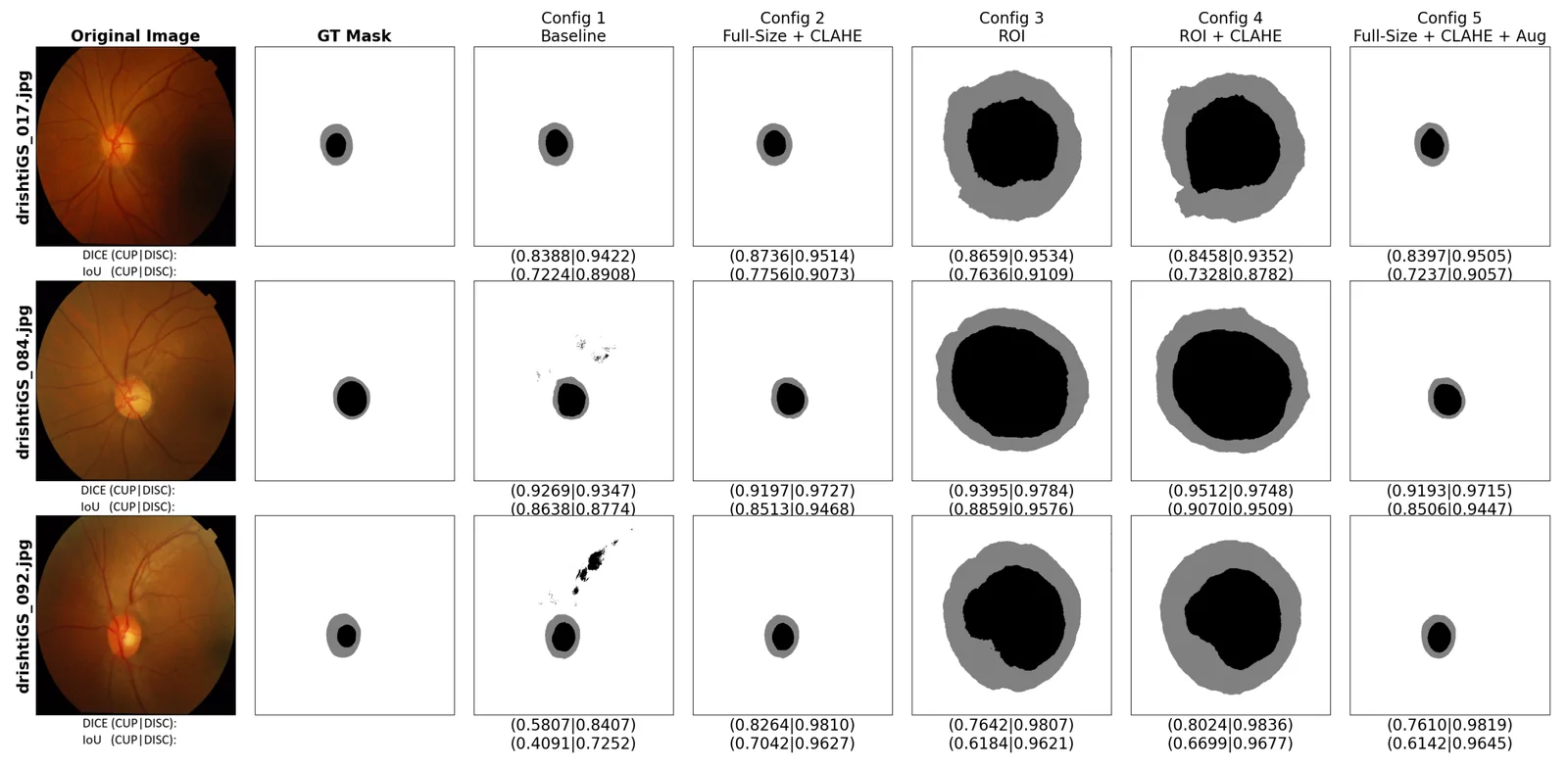
A representative Drishti-GS validation image under all five configurations, seed 42. Every configuration converges to a usable segmentation once the corrected training pipeline is used. Per-panel annotations give mean Dice across seeds; the full multi-seed summary is in Table 6.

Config 4 (ROI+CLAHE) delivered the best mean disc performance (0.9501±0.0088), while Config 5 led for the cup (0.8441±0.0389). Notable, however, is that Config 5 also exhibited the widest seed-to-seed spread observed across the entire study (disc SD = 0.0231, cup SD = 0.0389), underscoring that, even with fourfold augmentation, a training set of only 81 images leaves models sensitive to initialization. Augmentation does appear to offer the most dependable route to strong cup Dice on this dataset and we still advise its inclusion for small corpora, but the evidence no longer supports describing it as an absolute requirement once the base training configuration is properly tuned. Visual predictions for representative Drishti-GS samples are provided in Figure 4.

### 4.5. Convergence Summary Across Seeds

Table 7 lists the epoch of peak validation Dice for every configuration–seed pair. The spread within a single configuration is the striking part. Config 3 on REFUGE peaked at epoch 10 under seed 42, epoch 48 under seed 15, and epoch 22 under seed 89, a 38-epoch gap produced by nothing but a change of initialization.

Despite this variability, all 15 configuration–dataset combinations converged within the allocated 100-epoch training budget, and none exhibited the training collapse observed in the preliminary uncorrected experiments.

### 4.6. Comparison with Prior Literature

Table 8 summarizes representative results reported in prior studies for contextual comparison. Direct comparison should be interpreted cautiously due to differences in dataset splits, preprocessing pipelines, augmentation strategies, backbone architectures, and evaluation protocols.

Relative to these prior results, our best REFUGE configuration (Config 5, disc Dice 0.9523, cup Dice 0.8348) compares favourably to the modified U-Net with edge detection of [21] (disc 0.9700, cup 0.9000), though the latter incorporates additional edge-dilation postprocessing not included in our pipeline. On ORIGA, our Config 4 achieves the highest disc Dice (0.9681) and Config 5 the highest cup Dice (0.8873), both exceeding the M-Net baseline [12] (disc 0.9255, cup 0.8452) and the modified U-Net results [21] (disc 0.9600, cup 0.8700). Our Drishti-GS result trails the UT-Net transformer-based model [13], which is architecturally more sophisticated than the convolutional EfficientUNet++ used here. This study is deliberately framed as a controlled preprocessing ablation under a fixed architecture; its contribution is in characterizing which preprocessing choices matter, how much, and how reliably across seeds.

### 4.7. Computational Efficiency

Wall-clock training time, average inference throughput (FPS), and peak GPU memory usage were measured for each experiment. GPU memory usage remained approximately constant (≈13 GB) across all experiments, as the EfficientNet-B7 backbone accounted for the majority of the memory footprint. All configurations achieved an inference throughput exceeding 12 FPS, demonstrating that the proposed framework is computationally practical. Config 5 cost roughly three times the wall-clock training time of the unaugmented runs, which follows from the 4× expansion of its effective training set. Configs 3 and 4 ran somewhat faster, since a cropped region is cheaper to process.

### 4.8. Training Loss Convergence

**Figure 5:**
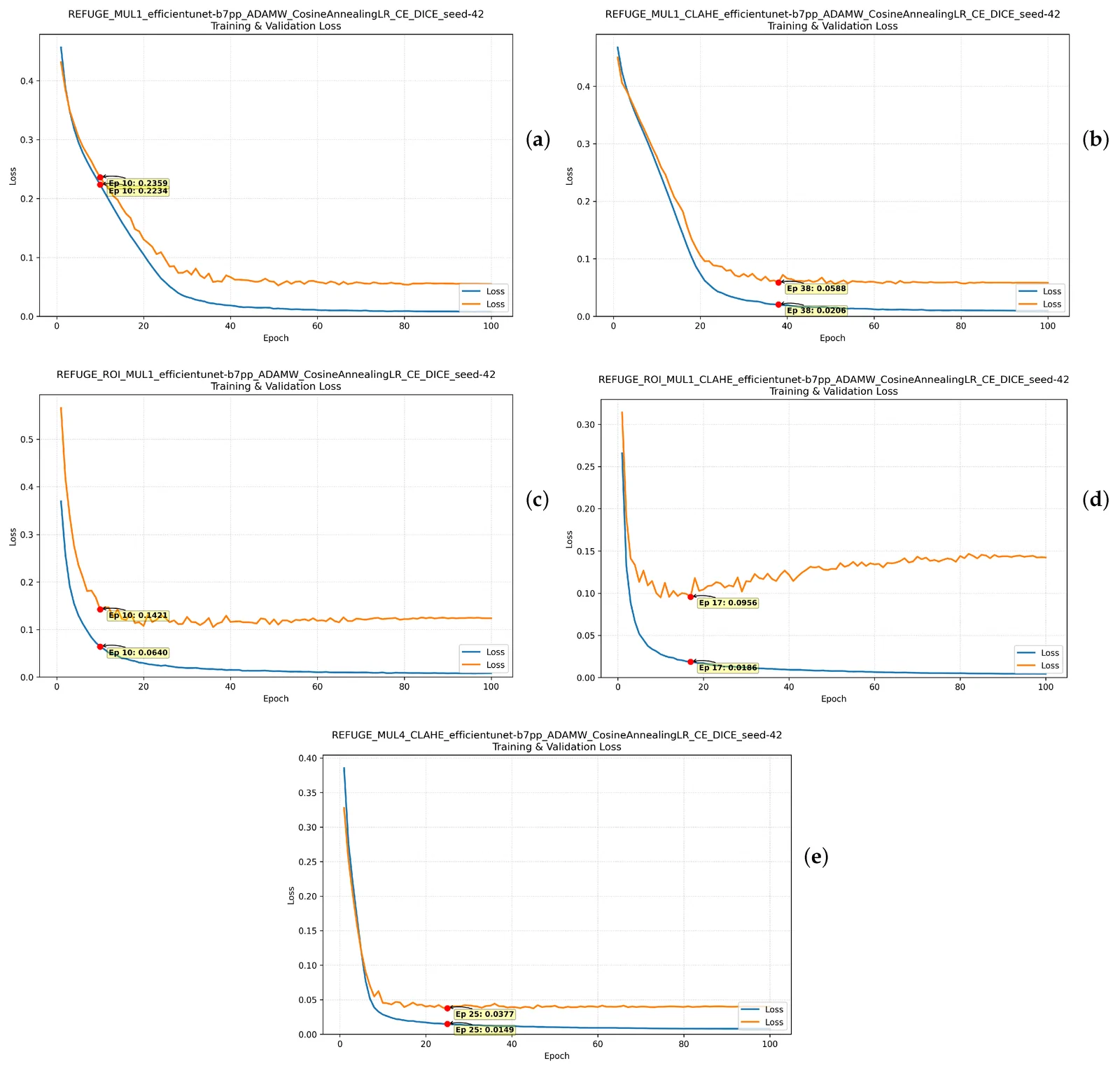
Training (blue) and validation (orange) loss curves for all five preprocessing configurations on the REFUGE dataset (seed 42). (**a**) Config 1 (Baseline); (**b**) Config 2 (Full+CLAHE); (**c**) Config 3 (ROI); (**d**) Config 4 (ROI+CLAHE); (**e**) Config 5 (Full+CLAHE+Aug). The red dot marks the best-performing epoch selected for evaluation. All five configurations converge smoothly within the 100-epoch budget. Complete curves for all datasets and seeds are available on Zenodo at https://doi.org/10.5281/zenodo.21416763.

Figure 5 shows training and validation loss curves for all five preprocessing configurations on the REFUGE dataset under seed 42 (as an example). All configurations converge smoothly and monotonically within the 100-epoch budget; the red dot in each panel marks the best-performing epoch retained for evaluation. Full loss curves for all datasets and all seeds are available in the Zenodo archive at https://doi.org/10.5281/zenodo.21416763.

### 4.9. Validation Dice and IoU Curves

**Figure 6:**
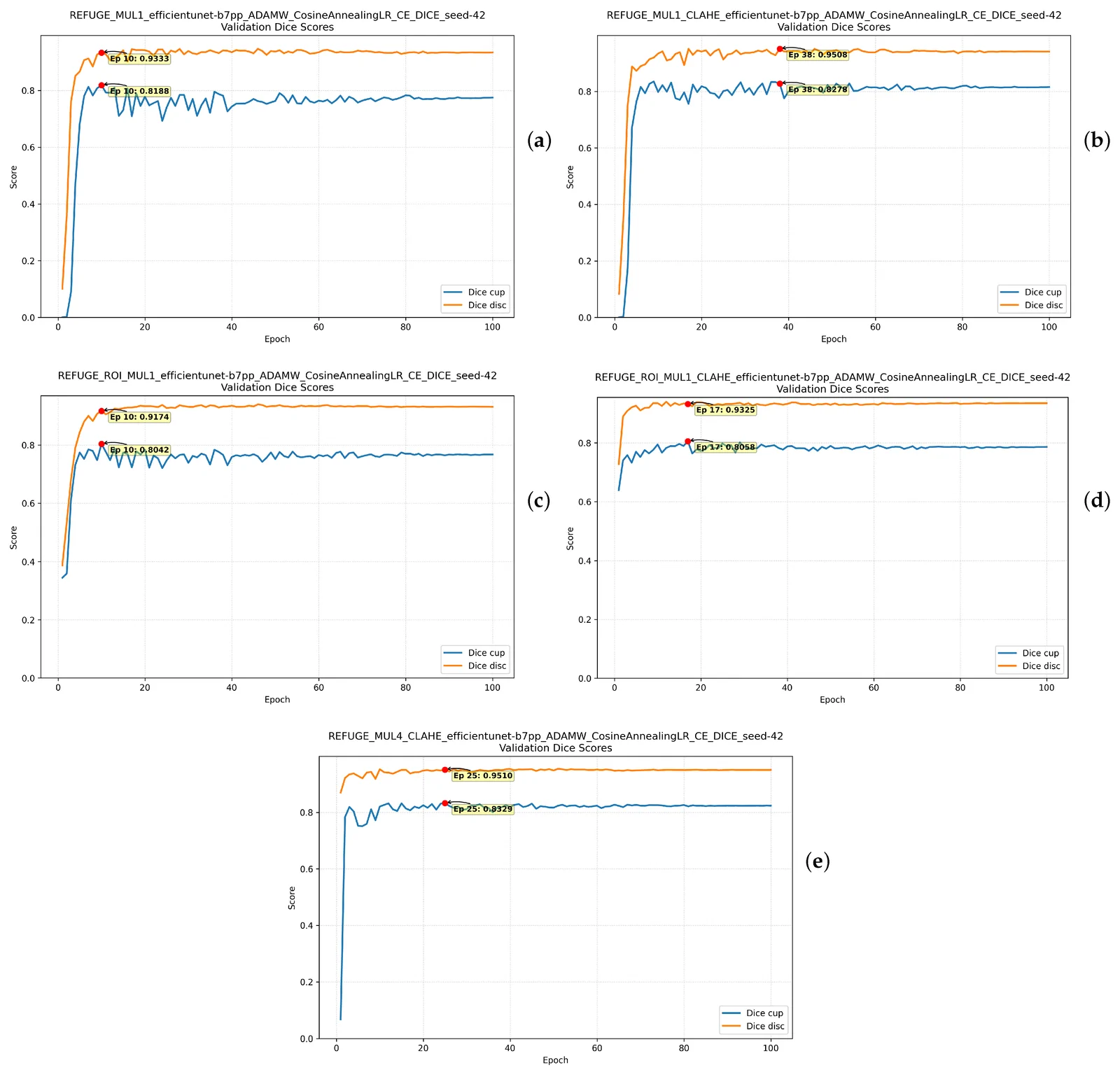
Validation Dice curves (optic disc and optic cup) across 100 training epochs for all five preprocessing configurations on the REFUGE dataset (seed 42). (**a**) Config 1 (Baseline); (**b**) Config 2 (Full+CLAHE); (**c**) Config 3 (ROI); (**d**) Config 4 (ROI+CLAHE); (**e**) Config 5 (Full+CLAHE+Aug). The red dot marks the best-performing epoch. IoU values follow the same trend and are derived from the Dice coefficients via IoU=Dice/(2−Dice). Complete curves for all datasets and seeds are available on Zenodo at https://doi.org/10.5281/zenodo.21416763.

Figure 6 shows validation Dice coefficients (optic disc and optic cup separately) across 100 training epochs for all five configurations on the REFUGE dataset under seed 42 (as an example). Each panel marks the best-performing epoch with a red dot. The IoU values follow the same trend as the Dice curves and are derived via IoU=Dice/(2−Dice) as described in Section 3.6. Complete validation Dice, IoU, precision, and recall curves for all datasets and all seeds are available in the Zenodo archive at https://doi.org/10.5281/zenodo.21416763.

### 4.10. Worst-Case Analysis

**Figure 7:**
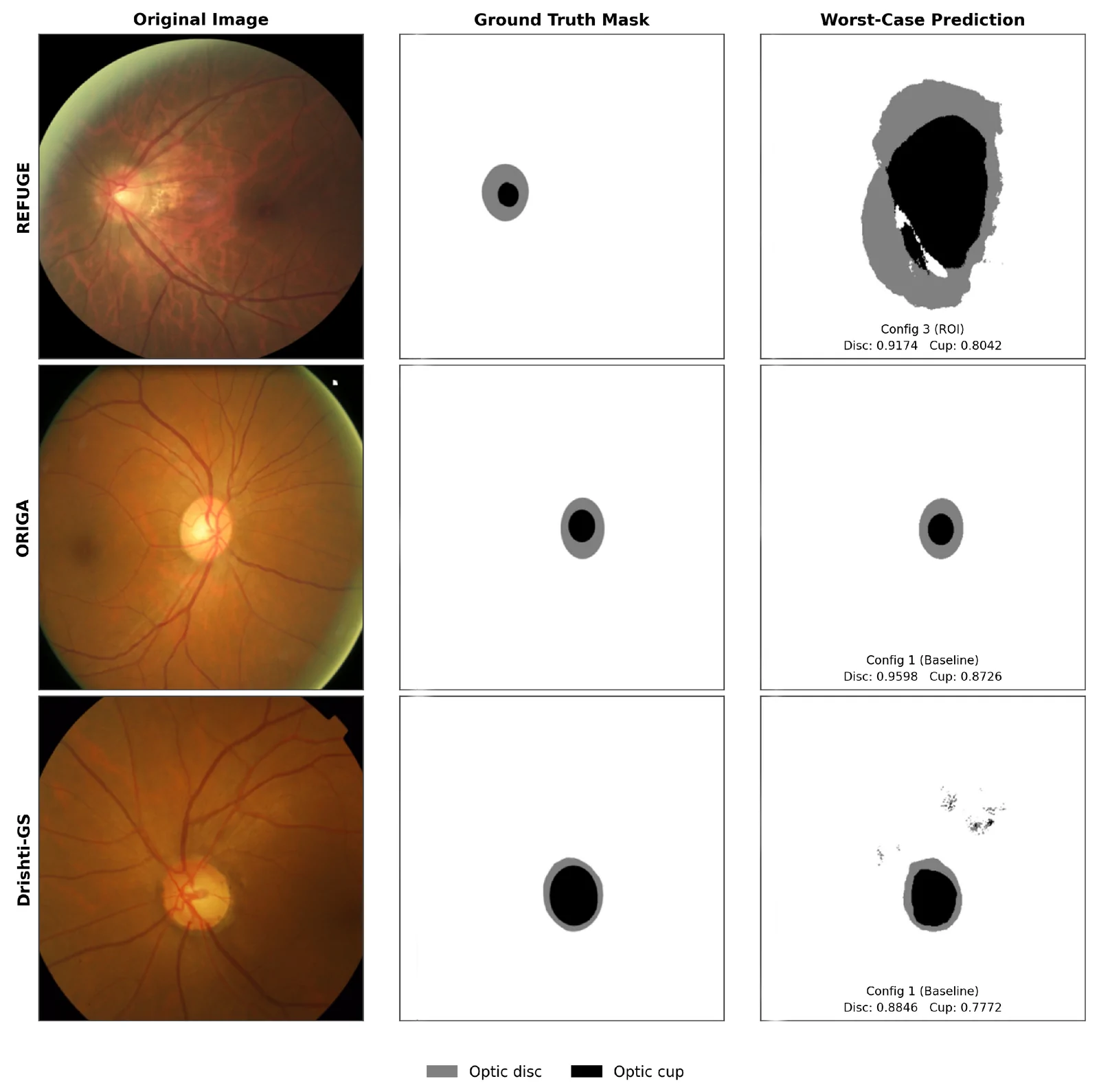
Worst-case segmentation results per dataset. Each row shows a representative validation sample from the lowest-performing preprocessing configuration identified from aggregate validation Dice (Table 7). REFUGE: Config 3 (ROI, seed 42; disc Dice 0.9174, cup Dice 0.8042) produces an oversized and boundary-misaligned prediction, consistent with the loss of peripheral context when cropping without contrast normalization. ORIGA: Config 1 (Baseline, seed 89; disc Dice 0.9598, cup Dice 0.8726) still yields a visually reasonable prediction; the narrow inter-configuration spread on ORIGA means the worst case remains within an acceptable performance range. Drishti-GS: Config 1 (Baseline, seed 15; disc Dice 0.8846, cup Dice 0.7772) produces scattered false-positive blobs outside the disc region, reflecting initialization sensitivity on only 81 training images.

Because the evaluation pipeline retains an aggregate validation Dice (Section 3.5), we report the worst-performing preprocessing configuration for each dataset, the configuration–seed pair that produced the lowest aggregate validation Dice, as an indicator of the lower-bound performance of the proposed pipeline. Figure 7 shows representative predictions from these worst-performing configurations.

On REFUGE, the worst result was produced by Config 3 (ROI cropping without CLAHE) under seed 42, with disc Dice 0.9174 and cup Dice 0.8042. This is the only configuration–seed combination on REFUGE to fall below 0.92 for disc segmentation, consistent with the reduced contextual information available when the peripheral retinal field is discarded without accompanying contrast normalization.

On ORIGA, all five configurations performed strongly. The weakest result was Config 1 (Baseline) under seed 89, with disc Dice 0.9598 and cup Dice 0.8726. The narrow gap between worst and best configurations (0.0083 for disc) confirms that ORIGA’s larger and more consistently acquired training set produces robust segmentation regardless of preprocessing choice.

On Drishti-GS, the worst result was Config 1 (Baseline) under seed 15, with disc Dice 0.8846 and cup Dice 0.7772. Even this worst-case result represents successful convergence—in contrast to the near-total failures (disc Dice < 0.05) observed in preliminary single-seed experiments with an uncorrected training setup, confirming that the corrected configuration eliminates catastrophic failure modes across all seeds.

## 5. Discussion

### 5.1. Interpretation of REFUGE Findings

On REFUGE, full-image CLAHE beat every ROI variant under all three seeds, whether or not augmentation was added. The likely mechanism is straightforward: normalizing contrast over the whole fundus gives the encoder usable gradients across the entire retinal field, and cropping throws that peripheral information away. Equally worth noting is how little Configs 2 and 5 moved between seeds, with the disc and cup SD both at or below 0.005. The advantage is reproducible, not the product of one fortunate initialization. It should be noted, however, that this finding was evaluated only with EfficientNet-B7; whether lighter or heavier encoders respond similarly to contrast normalization remains to be established and is noted as a limitation in Section 6.

### 5.2. Interpretation of ORIGA and Drishti-GS Findings

ORIGA inverts that. Here the ROI pipelines held a small advantage over their full-image counterparts and held it stably; Config 4’s disc SD is 0.0002. Focusing the network on the disc region appears to pay off modestly once the training set is large and the acquisition quality is uniform. Nevertheless, the overall difference across all five configurations is only about 0.0067 Dice units for disc segmentation, suggesting that architectural capacity, rather than the choice of preprocessing strategy, becomes the dominant factor once the training data are sufficiently large and consistently acquired.

The findings on Drishti-GS prompt a reassessment of the widely held view that data augmentation is indispensable for small medical imaging datasets. Repeated experiments across multiple random seeds under the corrected training setup show that all five preprocessing strategies are capable of converging. The key question is therefore not whether augmentation is required to prevent training failure, but whether it meaningfully improves performance and reduces variability. Overall, the results indicate that it does, particularly for cup segmentation, and we therefore retain our recommendation to include augmentation by default. What the evidence here will not support is the stronger claim that dropping augmentation causes collapse.

### 5.3. Computational Feasibility

Computational feasibility and clinical readiness are separate questions and should not be run together. A throughput above 12 FPS at roughly 13 GB of GPU memory means these pipelines could be run at a population-screening scale. It does not mean they should be. A credible deployment case would additionally require validation on cameras absent from the training sets, a systematic account of failure modes, calibrated uncertainty estimates, and prospective comparison against the screening pathway already in use. None of that is attempted here; Section 6 says more.

## 6. Limitations

The following limitations should be considered when interpreting the reported results.

Oracle ROI localization: The centre of each region-of-interest crop was determined from the centroid of the ground-truth annotation rather than from an independent optic disc detector. As a result, the reported ROI-based performance represents an optimistic upper bound on what a fully automated pipeline, which must first localize the optic disc before segmenting it, can realistically achieve.

Limited number of random seeds: Using three random seeds per configuration provides a practical measure of ranking stability but does not offer sufficient statistical power for formal hypothesis testing. In particular, the two-sided Wilcoxon signed-rank test requires at least five paired observations to achieve p<0.05. Consequently, the analysis is limited to descriptive statistics (mean ± SD) rather than inferential statistical testing.

Absence of cross-dataset transfer experiments: Models trained on one benchmark were not evaluated directly on another without fine-tuning. Consequently, the extent to which the observed preprocessing rankings generalize across different acquisition domains remains unknown.

Fixed CLAHE hyperparameters: The clip limit (8.0) and tile grid (8×8) were fixed throughout all experiments. Whether different CLAHE parameter settings would change the relative performance of the CLAHE-based configurations, particularly for datasets with different baseline contrast characteristics, was not investigated. A systematic sensitivity analysis varying the clip limit and tile grid size was not performed. Given that the performance advantage of CLAHE-based configurations over the baseline is the central finding on REFUGE, the possibility that a different parameter setting could alter this advantage, or that the fixed parameters are suboptimal for ORIGA and Drishti-GS, cannot be excluded. This limitation should be addressed in future work through a systematic sensitivity analysis.

Evaluation using a single backbone: All experiments were conducted with the EfficientNet-B7 backbone. Consequently, the reported preprocessing effects may not generalize to lighter or heavier backbones, and a systematic comparison across the EfficientNet family remains an avenue for future work. In particular, it should not be assumed that the CLAHE advantage observed here would carry over to lighter encoders (e.g., EfficientNet-B0 through B3), which may respond differently to contrast normalization. Evaluating whether these preprocessing trends are consistent across different backbone sizes and architectures is an important direction for future work.

Literature comparison is not a controlled head-to-head benchmark: The studies summarized in Table 8 differ in terms of training splits, loss functions, data augmentation strategies, and evaluation protocols. They are therefore intended to provide contextual reference points rather than fair comparisons under identical experimental conditions.

## 7. Conclusions

We have presented a controlled, multi-seed ablation of five preprocessing pipelines for optic disc and cup segmentation, benchmarked using EfficientUNet++ on the REFUGE, ORIGA, and Drishti-GS datasets. Repeating each experiment across three independent random seeds was the central methodological decision of the study and it materially changed several conclusions that a single-run analysis would have drawn. Most significantly, the near-total training failure of unaugmented configurations on Drishti-GS, which a preliminary uncorrected run suggested, did not recur once the optimizer settings and contrast normalization were corrected, shifting the explanation from data scarcity to training configuration sensitivity.

From the multi-seed results, three practical recommendations emerge. First, applying CLAHE to full-resolution images (clip limit 8.0, tile grid 8 × 8, V-channel of HSV colour space) is the single most consistently beneficial preprocessing step across all three datasets and acquisition conditions, and is advisable as a default. Second, on larger and more homogeneously acquired datasets such as ORIGA, ROI cropping was associated with a small, initialization-stable advantage in our experiments, though the absolute gap across all five configurations (0.0067 Dice) is narrow enough to suggest that architectural capacity, rather than preprocessing choice, is the primary determinant of performance once training data are abundant (Section 5). Practitioners should treat the ROI+CLAHE advantage as a minor, dataset-conditional trend rather than a strong recommendation, and must additionally account for the fact that the ROI localization in this study was derived from ground-truth annotations; a real deployment would require an independent disc detector and the resulting performance may differ from that observed under the ground-truth-based localization setting.

Priorities for future work include: replacing the oracle ROI with a prospectively evaluated disc localization model; instrumenting the evaluation pipeline to retain per-image Dice scores so that proper paired significance tests can be run; conducting cross-dataset transfer experiments to assess how robust the identified preprocessing rankings are across acquisition domains; and performing a systematic sensitivity analysis of CLAHE hyperparameters, including clip limit and tile grid size, as well as EfficientNet backbone sizes.

## Figures and Tables

**Table 1:** Summary of datasets used in this study.

Dataset	Images	Resolution	Train	Val	Glaucoma %	Quality
REFUGE [18]	400	2124 × 2056	320	80	10.0%	High, consistent
ORIGA [19]	650	3072 × 2048	520	130	25.8%	Variable
Drishti-GS [20]	101	2896 × 1944	81	20	69.3%	Diverse

**Table 2:** Experimental preprocessing configurations. All other training variables held constant.

ID	Name	Full	ROI	CLAHE	Aug
Config 1	Baseline	✔	—	—	—
Config 2	Full-Size + CLAHE	✔	—	✔	—
Config 3	ROI	—	✔	—	—
Config 4	ROI + CLAHE	—	✔	✔	—
Config 5	Full-Size + CLAHE + Aug	✔	—	✔	4×

**Table 3:** EfficientNet-B7 encoder stage specifications and UNet++ decoder connections used in this study. Values verified from the actual encoder using a 512 × 512 input. Skip connections bridge each encoder stage to the corresponding decoder level via nested dense pathways.

Stage	Output Resolution	Channels	Blocks	Skip to Decoder Level
1	256 × 256	64	2	Level 1
2	128 × 128	48	7	Level 2
3	64 × 64	80	7	Level 3
4	32 × 32	224	10	Level 4
5	16 × 16	640	17	Level 5
Segmentation head				3-class softmax (background, cup, disc)

**Table 4:** Overall segmentation performance on REFUGE. Mean ± SD across three random seeds (42, 15, 89). Best result per column in bold. ^†^ IoU derived from mean Dice via IoU=Dice/(2−Dice).

Configuration	Disc Dice (Mean ± SD)	Disc IoU ^†^	Cup Dice (Mean ± SD)	Cup IoU ^†^
Config 1 (Baseline)	0.9320±0.0056	0.8727	0.8014±0.0200	0.6686
Config 2 (Full+CLAHE)	0.9465±0.0038	0.8984	0.8230±0.0050	0.6992
Config 3 (ROI)	0.9297±0.0107	0.8686	0.7931±0.0100	0.6571
Config 4 (ROI+CLAHE)	0.9351±0.0047	0.8781	0.8014±0.0038	0.6686
Config 5 (Full+CLAHE+Aug)	0.9523±0.0017	**0.9089**	0.8348±0.0018	**0.7164**

**Table 5:** Overall segmentation performance on ORIGA. Mean ± SD across three random seeds (42, 15, 89). Best result per column in bold. ^†^ IoU derived from mean Dice via IoU=Dice/(2−Dice).

Configuration	Disc Dice (Mean ± SD)	Disc IoU ^†^	Cup Dice (Mean ± SD)	Cup IoU ^†^
Config 1 (Baseline)	0.9614±0.0017	0.9257	0.8744±0.0016	0.7768
Config 2 (Full+CLAHE)	0.9650±0.0002	0.9324	0.8775±0.0013	0.7817
Config 3 (ROI)	0.9666±0.0003	0.9354	0.8796±0.0011	0.7851
Config 4 (ROI+CLAHE)	0.9681±0.0002	**0.9382**	0.8837±0.0010	0.7916
Config 5 (Full+CLAHE+Aug)	0.9652±0.0006	0.9327	0.8873±0.0024	**0.7974**

**Table 6:** Overall segmentation performance on Drishti-GS. Mean ± SD across three random seeds (42, 15, 89). Best result per column in bold. ^†^ IoU derived from mean Dice via IoU=Dice/(2−Dice).

Configuration	Disc Dice (Mean ± SD)	Disc IoU ^†^	Cup Dice (Mean ± SD)	Cup IoU ^†^
Config 1 (Baseline)	0.9084±0.0237	0.8322	0.7955±0.0158	0.6604
Config 2 (Full+CLAHE)	0.9393±0.0078	0.8855	0.8111±0.0379	0.6822
Config 3 (ROI)	0.9368±0.0139	0.8811	0.7745±0.0043	0.6319
Config 4 (ROI+CLAHE)	0.9501±0.0088	**0.9049**	0.7793±0.0042	0.6384
Config 5 (Full+CLAHE+Aug)	0.9431±0.0231	0.8923	0.8441±0.0389	**0.7303**

**Table 7:** Best validation epoch achieved for each random seed (42/15/89) across all datasets and preprocessing configurations. Differences in the selected epoch within a configuration reflect sensitivity to weight initialization, whereas all configurations converged within the 100-epoch training budget.

Dataset	Configuration	Best Epoch (42/15/89)	Disc SD
REFUGE	Config 1 (Baseline)	10/29/41	0.0056
	Config 2 (Full+CLAHE)	39/16/48	0.0038
	Config 3 (ROI)	10/48/22	0.0107
	Config 4 (ROI+CLAHE)	17/20/26	0.0047
	Config 5 (Full+CLAHE+Aug)	25/38/56	0.0017
ORIGA	Config 1 (Baseline)	85/60/88	0.0017
	Config 2 (Full+CLAHE)	42/32/65	0.0002
	Config 3 (ROI)	26/57/44	0.0003
	Config 4 (ROI+CLAHE)	24/25/44	0.0002
	Config 5 (Full+CLAHE+Aug)	58/83/46	0.0006
Drishti-GS	Config 1 (Baseline)	21/18/36	0.0237
	Config 2 (Full+CLAHE)	17/33/31	0.0078
	Config 3 (ROI)	17/26/67	0.0139
	Config 4 (ROI+CLAHE)	38/28/37	0.0088
	Config 5 (Full+CLAHE+Aug)	8/9/38	0.0231

**Table 8:** Contextual reference points from prior studies on REFUGE, ORIGA, and Drishti-GS. These values are not directly comparable to those of the present study and are included solely to situate the present results within the broader literature. Results from prior studies are reported as presented in the original sources. “This study” rows reporting best disc and best cup separately reflect different best-performing configurations for each metric and do not represent a single configuration achieving both values simultaneously.

Dataset	Method	Disc Dice	Cup Dice	Source
REFUGE	Modified U-Net + edge detection & dilation	0.9700	0.9000	[21]
REFUGE	UT-Net (U-Net + Transformer)	0.9593	0.9267	[13]
REFUGE	EfficientUNet++, Config 5 (Full+CLAHE+Aug)	0.9523	0.8348	This study
ORIGA	M-Net with polar transformation	0.9255	0.8452	[12]
ORIGA	Modified U-Net + edge detection & dilation	0.9600	0.8700	[21]
ORIGA	EfficientUNet++, Config 4 (ROI+CLAHE)—best disc	0.9681	—	This study
ORIGA	EfficientUNet++, Config 5 (Full+CLAHE+Aug)—best cup	—	0.8873	This study
Drishti-GS	UT-Net (U-Net + Transformer)	0.9792	0.9409	[13]
Drishti-GS	EfficientUNet++, Config 4 (ROI+CLAHE)—best disc	0.9501	—	This study
Drishti-GS	EfficientUNet++, Config 5 (Full+CLAHE+Aug)—best cup	—	0.8441	This study

## Data Availability

The REFUGE, ORIGA, and Drishti-GS datasets analysed in this study are publicly available (see [18,19,20]). The training and evaluation code produced for this study is available at https://github.com/mohamedsafy/src (accessed on 2 September 2026). The experimental results archive, including training and validation loss curves, metric curves, and prediction progression matrices for all five configurations and three seeds across all three datasets, is openly available on Zenodo at https://doi.org/10.5281/zenodo.21416763.

## Abbreviations

The following abbreviations are used in this manuscript:

AMP	Automatic Mixed Precision
CDR	Cup-to-Disc Ratio
CLAHE	Contrast Limited Adaptive Histogram Equalization
DSC	Dice Similarity Coefficient
IoU	Intersection over Union
ROI	Region of Interest
SD	Standard Deviation
